# Confronting the public health challenge of inaccessible COVID-19 home tests: insights from the RADx® Tech Accessibility Initiative

**DOI:** 10.3389/fpubh.2025.1586514

**Published:** 2025-06-23

**Authors:** Emily B. Kennedy, Kimberly Noble, Kevin Leite, Maren Downing, Samuel Dolphin, Mia Cirrincione, Brian Walsh

**Affiliations:** ^1^OOMVELT, LLC, Lakewood, OH, United States; ^2^Kimberly Noble Consulting, LLC, Hermosa Beach, CA, United States; ^3^Leite Consulting LLC, Holly Springs, NC, United States; ^4^M Biomedical LLC, McCormick, SC, United States; ^5^Independent Researcher, Cambridge, MA, United States; ^6^MJC Innovation LLC, Chicago, IL, United States; ^7^Innova Group, LLC, Alpharetta, GA, United States

**Keywords:** accessibility, aging population, COVID-19, disability, human factors, *in vitro* diagnostics, medical devices, product design

## Abstract

COVID-19 home tests first distributed by the U.S. Government in early 2022 proved inaccessible to Americans with no vision, low vision, limited dexterity, and certain aging-related impairments. The National Institute of Biomedical Imaging and Bioengineering (NIBIB) leveraged a multimillion-dollar investment to address this challenge via the Rapid Acceleration of Diagnostics (RADx®) Tech Accessibility Initiative. What follows is a case study report on the March 2022–June 2023 implementation of this initiative, which was later expanded into a larger-scale program called RADx Tech III (September 2022–present). The initiative unveiled a substantial gap in resources guiding accessible product development and applied crisis response funding to bridge this gap. Beyond the primary goal of improving accessibility of COVID-19 home tests, the initiative was successful in developing an expert resource pool, documenting best practices for design of accessible home tests, and generating/validating a framework for future accessibility initiatives.

## 1 Introduction

In January 2022, the U.S. Government began distributing rapid COVID-19 home tests to American households. Stand-up of this program required the unprecedented purchase of a billion tests, development of high-capacity online and phone ordering platforms, and close coordination with the U.S. Postal Service for rush delivery. The Administration took notable steps to ensure this program reached hard-hit and high-risk communities by prioritizing order processing for vulnerable households (1, 2). Despite best intentions to promote broad and equitable access, the COVID-19 home tests distributed to Americans proved inaccessible to individuals with no vision, low vision, limited dexterity, and certain aging-related impairments. Soon after this government program launched, disability and senior citizen advocacy groups called for distribution of more accessible tests. In February 2022, the National Institute of Biomedical Imaging and Bioengineering (NIBIB) was activated through a multimillion-dollar investment to respond quickly to concerns about the accessibility of COVID-19 home tests. To expedite impact, NIBIB strategically targeted incremental innovation of products already funded via the institute’s preexisting Rapid Acceleration of Diagnostics (RADx®) Tech program, launched at the start of the pandemic to increase national COVID-19 testing capacity (3–5).

What follows is a case study report on the March 2022–June 2023 implementation of NIBIB’s RADx Tech Accessibility Initiative (hereafter termed “Initiative”). Personnel committed to the Initiative’s primary objective of improving accessibility of COVID-19 home tests learned quickly that there were few, if any, resources to guide accessible product development, and the Initiative was uniquely positioned to bridge this resource gap. COVID-19 crisis response funding provided a rare opportunity to assemble the extensive resources required to develop and validate a comprehensive framework for the innovation of accessible at-home diagnostics. The resulting framework, distilled in *Best Practices for the Design of Accessible COVID-19 Home Tests* (6), has significant potential to advance accessibility in other areas of the consumer medical device industry. This case study report details its development to increase awareness and inspire broader application.

Improving accessibility of home use medical devices is crucial because relying on others to use these products compromises health privacy and can result in treatment delays if a caregiver is not affordable or immediately available. A core belief shared by advocacy groups is that all people, regardless of age or disability, should be empowered with maximum opportunity to live autonomous, functionally independent lives. Design modifications to enhance product accessibility can facilitate independent use by a large fraction of the U.S. population. An estimated 13.5% of working-age Americans (18–64 years old) (7) and 43.9% of older adults (65 years or older) live with at least one disability (8). Many are members of the specific subpopulations targeted by this Initiative. Among all adults in the U.S. (18 years or older), at least 5.5% live with no vision or low vision (9), 7% with limited dexterity (10), and 13.9% with cognitive impairments often associated with aging (9). Making a product more accessible for any of these groups tends to improve the user experience for everyone, regardless of ability.

## 2 Initiative resourcing

### 2.1 Listening session

The Initiative kicked off in March 2022 with a listening session. The purpose of this session was to solicit input from advocacy groups and government agencies on pain points associated with current tests and potential near-term and long-term solutions. Common observations and key takeaways informed Initiative priorities. These included the need for more intuitive test packaging, better-organized package contents, easier to handle test components, minimal fluid handling, clearer instructions, and simpler workflows. The listening session also served to open lines of communication for ongoing discussion and partnership with key stakeholders (11).

### 2.2 Review of design standards and guidelines

A literature search was conducted to identify pre-existing accessibility-related *in vitro* diagnostic (IVD) design standards or guidelines that could inform Initiative design and development. None were identified, so the team broadened the scope of the search to standards or guidelines for the larger category of consumer medical devices, and then further to consumer products in general. Still, no formal standards for accessible product design were identified. This finding was corroborated via interviews with advocacy groups, government agencies, and engineering design groups. Several guidelines relevant to COVID-19 home test design, such as the World Wide Web Consortium’s (W3C®) Web Content Accessibility Guidelines (WCAG) 2.2 (12), Apple’s Human Interface Guidelines for Accessibility (13), and The Centre for Excellence in Universal Design’s (CEUD) 7 Principles (14), were identified, but these were limited in scope. The absence of sufficient foundational material meant the framework for the Initiative needed to be constructed anew.

### 2.3 COVID-19 home test manufacturers

To make COVID-19 home tests more accessible, buy-in from test manufacturers was essential. All active NIBIB-funded RADx Tech portfolio companies developing or scaling innovative, rapid COVID-19 home tests were invited to participate in the Initiative. Invitees included manufacturers of both antigen- and molecular-based technologies, visually read and reader-assisted technologies, traditional lateral flow assays, and other technology types. Some of these manufacturers had products which had already achieved FDA Emergency Use Authorization (EUA), but most were still seeking authorization. Ultimately, technologies manufactured by 15 of a total 24 invited companies underwent evaluation. Several manufacturers declined to participate due to resource constraints or perceived internal capability. Several other evaluations were deferred by Initiative leadership after validation testing revealed technological immaturity.

### 2.4 Accessibility consultants

A pre-existing RADx Tech partnership with The Georgia Institute of Technology’s HomeLab (hereafter termed “HomeLab”), situated in the institute’s Center for Advanced Communications Policy, was leveraged to secure accessibility expertise. HomeLab is an independent testing facility staffed by scientists and engineers trained to systematically evaluate the usability and accessibility of products that promote independent living. One of HomeLab’s core competencies is influencing the design of technologies that promote health, wellness, and independence for older adults (15). HomeLab had ample experience with COVID-19 tests, since it had been the designated resource for RADx Tech usability evaluations since April 2020. The Initiative engaged HomeLab in April 2022.

Recognizing the critical importance of involving representatives of target user groups in product evaluations (16), in addition to academic experts, the Initiative sought subject matter expert (SME) consultant referrals from advocacy groups and government agencies that had attended the March 2022 listening session. Candidates were down selected via structured interviews, with the Initiative contracting its first SMEs in May 2022. At peak, 18 SMEs–15 individuals living with disability or aging-related impairments and three with deep academic knowledge–were under contract in service of the Initiative. Subpopulation expertise included eight representatives for no vision, six for low vision, two for limited dexterity, and eight for aging. Two of the academics had expertise spanning all target user groups.

### 2.5 Design firms

Design firms were engaged to assist the Initiative with accessible COVID-19 home test concept generation and prototyping. Initiative personnel queried professional networks and the web to identify firms with experience designing accessible at-home diagnostics. Finding none, the team broadened the scope of the search to firms with experience designing handheld devices or consumables, especially for seniors or users with disabilities. Initiative personnel met with eight shortlisted firms to gauge fit and ultimately invited four firms with complementary skillsets to submit proposals for 2D/3D visualization of short-term (3-months) and medium-term (6–9 months) design improvement opportunities.

Design firm proposals submitted to the Initiative underwent a rigorous Vendor Review Panel (VRP) vetting process. VRP members researched vendors, conducted reference checks, reviewed proposals, and clarified specifics before making recommendations on whether to proceed with contracting (17). Final contracting decisions were made by NIBIB. This process ensured only high-caliber firms were selected. The Initiative contracted the first of four design firms in May 2022.

### 2.6 Stakeholder community

A standing monthly meeting series with advocacy groups and government agencies commenced in May 2022 to update the stakeholder community on Initiative activities and provide a forum for Initiative personnel to collect input. All the groups and agencies represented at the March 2022 listening session were invited to participate. At monthly meetings, stakeholders asked insightful questions and provided feedback that influenced the structure and trajectory of the Initiative, ensuring it best served the accessibility community at large.

## 3 Methods

**Figure 1:**
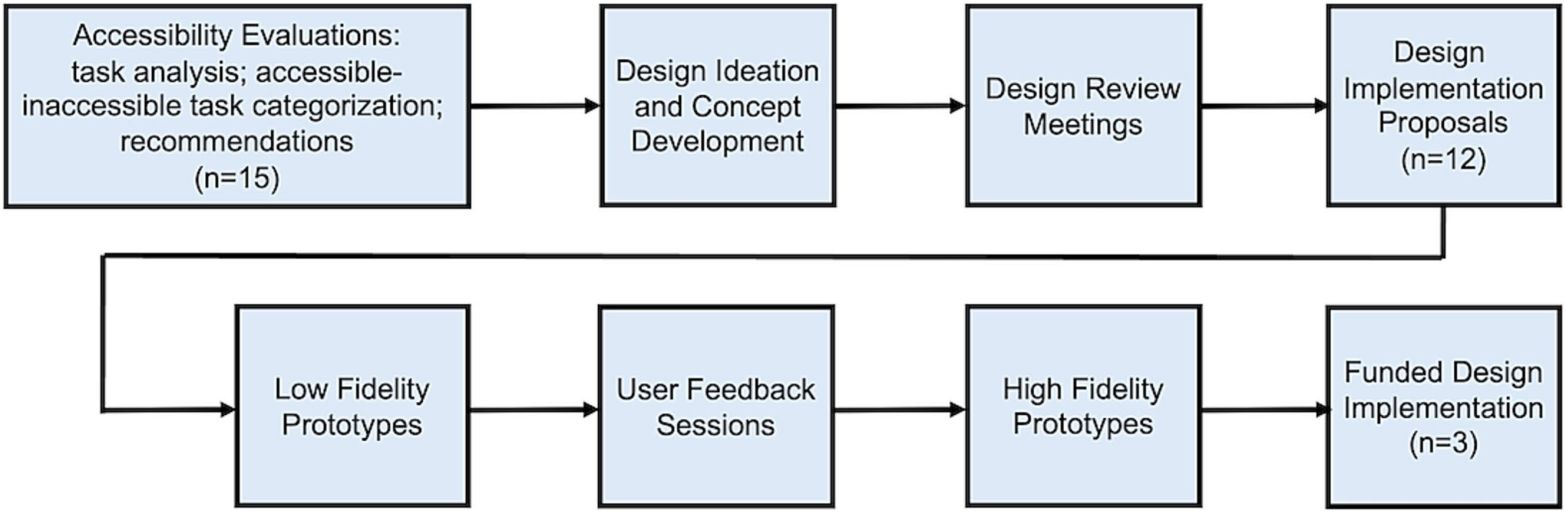
NIBIB RADx Tech Accessibility Initiative process where “*n*” represents the number of participating COVID-19 home test manufacturers at each phase.

Resources were activated to implement key Initiative processes (Figure 1), centering around evaluation and redesign of technologies to improve accessibility.

### 3.1 Technology prioritization schema

Technologies were prioritized for evaluation using metrics that identified prime targets for rapid redesign and deployment. Priority was given to technologies with highest baseline accessibility. Baseline accessibility was measured using Likert scale indications of the extent to which a technology embodied desired attributes voiced in the March 2022 listening session. These attributes included, but were not limited to, easy-open packaging, organized contents, easy-to-handle components, limited fluid handling, clear instructions, and simplified workflow. Technologies were also prioritized for evaluation based on regulatory status. Those that had already achieved FDA EUA were fast-tracked.

### 3.2 Accessibility evaluations

Between May 2022 and January 2023, a standard, stepwise evaluation process was applied to technologies in order of priority, wherein each product’s workflow was deconstructed into discrete tasks, tasks were categorized as accessible or inaccessible, and recommendations to improve ease of use or resolve inaccessible tasks were made. First, sample test kits were shipped to HomeLab where a multidisciplinary team of scientists and engineers systematically reviewed the test kit and instructions to conduct a hierarchical task analysis, breaking down the workflow into a list of every individual action comprising the end-to-end testing process from kit box opening through disposal. While quick reference instructions (QRI) for a traditional lateral flow assay rarely exceed 10 numbered steps, a single step within a QRI may consist of multiple, isolable actions. For example, a sample collection step could involve the discrete tasks of opening the swab packaging, inserting the swab into a nostril, rotating the swab, counting and/or timing rotations, and so forth. HomeLab’s task analysis dissected a typical, less-than-10-step QRI into more than 40 discrete tasks on average. Each discrete task was then categorized as either accessible or inaccessible to the four target user groups: no vision, low vision, limited dexterity, and aging. For each task categorized as inaccessible, the HomeLab team provided free response recommendations for how to render the task accessible. Recommendations were also made for tasks which were deemed accessible but potentially challenging for most users.

Next, SMEs–including individuals living with disabilities or aging-related impairments–were shipped sample test kits and provided HomeLab’s task list, without HomeLab’s accessible or inaccessible determinations, to use as a template for conducting their own accessibility evaluations. The SMEs then independently evaluated the accessibility of each task for users in their subpopulation(s) of expertise and provided recommendations for improvement. Individual SME evaluations were summarized into a single report of consensus accessible-inaccessible task categorizations, noted issues, and collective recommendations.

The HomeLab team and SMEs largely agreed on accessible-inaccessible task categorization, but their specific recommendations for making inaccessible tasks accessible varied. HomeLab recommendations routinely accounted for all four target user groups’ cross-cutting needs, whereas SME recommendations were often derived from personal experience and thus focused on the needs of the specific target user group(s) they represented. The result was recommendations appropriately balancing breadth and depth. The test kit manufacturer received both HomeLab’s report and the consolidated SME report.

### 3.3 Accessible design concept development and implementation

Design firms contracted by the Initiative were provided with sample test kits and copies of the HomeLab and SME accessibility evaluations. Design firms used these inputs to generate preliminary accessible design concepts. Concepts were reviewed by several SMEs and refined based on their feedback. Design firms then presented refined design concepts to respective test manufacturers in design review meetings that took place between June 2022 and March 2023. Meeting attendance by accessibility SMEs proved critical for bridging the design firms’ knowledge gap when fielding manufacturer questions about accessible design features.

Subsequently, Initiative personnel worked with manufacturers to assess readiness for implementation of design concepts. Manufacturers of 12 of the 15 products which had undergone accessibility evaluations and initial design sprints were enthusiastic about the near-term implementation of resulting concepts with continued Initiative support and oversight. Three manufacturers declined to participate further due to cost and complexity of solutions. Each of the remaining 12 COVID-19 home test manufacturers worked with Initiative personnel to prioritize accessible design concepts and develop a proposal for implementation, complete with project budget and timeline. Design firms developed low fidelity prototypes for evaluation in five user feedback sessions which took place between December 2022 and May 2023. In total, 152 accessible design concepts were evaluated by 94 target users, with collective feedback provided to respective test manufacturers. Three of the 12 proposals were ultimately funded by NIBIB. Funded manufacturers developed high-fidelity prototypes of preferred concepts for further evaluation prior to implementation.

## 4 Results

### 4.1 COVID-19 home test technological advances

**Figure 2:**
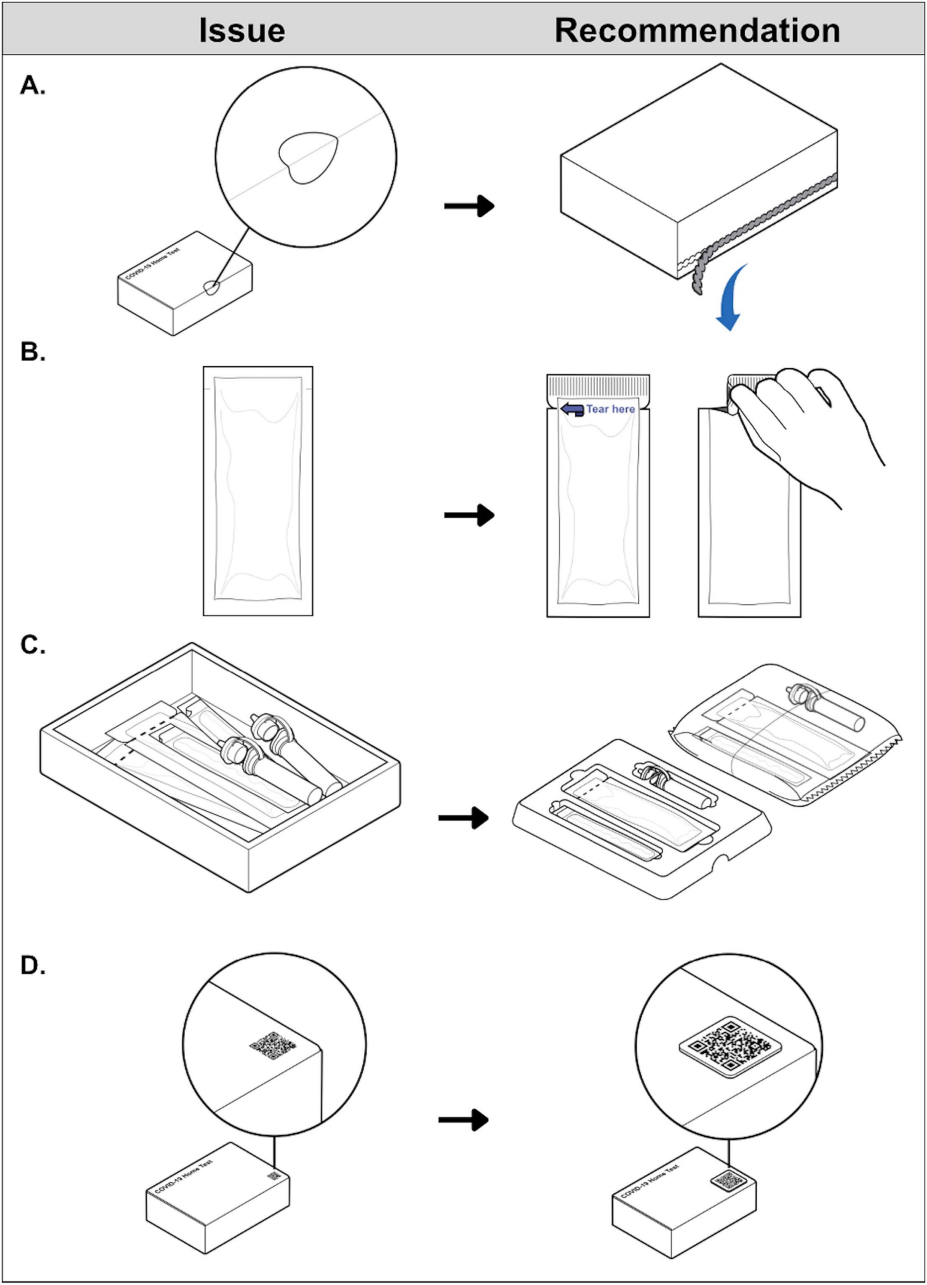
Examples of COVID-19 home test technological advances resulting from NIBIB’s RADx Tech Accessibility Initiative. Product features represent improvements to the accessibility of external packaging. **(A)** Adapted with permission from “Difficult to remove tamper-evident seal” by U.S Access Board, internal packaging. **(B)** Adapted with permission from “Pouch without clear indication for tear location” and “Pouch with evident tear locations” by U.S Access Board, test kit organization. **(C)** Adapted with permission from “Loose contents inside box packaging” and “Contents neatly organized in a tray or a tear pouch” by U.S Access Board, and test kit acquisition. **(D)** Adapted with permission from “Example of a flat QR” and “Alternative to flat QR code” by U.S Access Board.

The three funded manufacturers integrated a range of accessible design features into their commercial COVID-19 home test products’ instructions and packaging.^1^ For example, outer box labeling and printed instructions now use larger fonts and higher contrast ratios to improve legibility. Digital instruction PDFs are tagged for ease of reading, navigation, and recognition of warnings when using assistive technologies. Non-text media contained in digital instructions (e.g., illustrations and symbols) include descriptive alternative text, enabling equal access to visual content and facilitating nonvisual discernment of kit components. Instructions are now provided in alternative formats, such as closed-captioned video tutorials with extended audio description and full transcripts, and phone-based interactive voice response systems. This is advantageous to users who prefer to process information visually and/or audibly. Product websites have also been updated according to WCAG and SME feedback. Clear adhesive box seals, that some users were unable to open without scissors (potentially damaging internal components in the process), have been replaced with perforated flap closures that are easier to locate and disengage (Figure 2A). Two manufacturers implemented larger tear notches with high-contrast labels for internal pouches making the tear location easier to identify and grip (Figure 2B). One manufacturer added thumb cutouts to their kit organization trays to facilitate component removal using a familiar tactile cue (Figure 2C). Two manufacturers also have plans to incorporate tactile QR codes on outer packaging to digitally communicate kit expiration dates and link to resources, enabling more customers to rapidly acquire product information in a retail environment regardless of box labeling legibility (Figure 2D).

### 4.2 Documented best practices

Accessibility evaluations revealed many technologies had similar issues. In July 2022, the Initiative formed a working group to capture common issues and universal design recommendations, validated through user feedback sessions, in a best practices document. The purpose was to preserve learnings, preventing loss upon dissolution of the RADx Tech Program. In June 2023, the U.S. Access Board published the resultant *Best Practices for the Design of Accessible COVID-19 Home Tests* Technical Assistance Document (6). With NIBIB’s support, Initiative personnel are currently working with relevant domestic and international organizations to disseminate content of this document, which has potential for future adaptation into a formal accessible IVD design standard.

### 4.3 RADx Tech III Program

The success of NIBIB’s initial RADx Tech Accessibility Initiative (March 2022–June 2023), motivated follow-on funding for a larger-scale NIBIB program called RADx Tech III, launched in September 2022. In contrast to the preceding Initiative, which focused on making incremental and expedited improvements to the accessibility of mature COVID-19 testing technologies, RADx Tech III sponsored development of early-stage COVID-19 testing technologies with longer development timelines.

The RADx Tech III Program solicited proposals of two types between September and October 2022. The first call was for over the counter (OTC) COVID-19 diagnostic technologies capable of being implemented independently by users with no vision, low vision, limited dexterity, and certain aging-related impairments. Competitive responses described technologies with significantly improved accessibility and ease-of-use versus existing commercial platforms, and at least equal analytical and clinical performance (18). The second call was for the next generation of high-performance COVID-19 rapid tests. Here, competitive responses described technologies with significantly improved analytical and clinical performance, and at least marginally improved accessibility and ease of use (19). Notably, a vast majority of proposals indicated limited understanding of accessibility. Many recommended Braille as a one-dimensional solution despite associated challenges (20), and in lieu of more universal approaches.

The RADx Tech III Program received 220 project proposals. All proposals were reviewed by a Viability Panel for technical, clinical, regulatory, and commercialization feasibility to determine which should advance. Sixty-four (64) submissions, or approximately 30%, advanced to the next phase, a multi-week interactive vetting process. Findings were presented to a Steering Panel charged with making funding recommendations to NIBIB. Accessibility consultants who previously supported the Initiative, including SMEs living with a disability, were enlisted to serve as key voting members of the Steering Panel. In total, 27 of the initial 220 project proposals, or approximately 12%, were recommended by the Steering Panel and approved by NIBIB to receive funding through the RADx Tech III Program.

**Figure 3:**
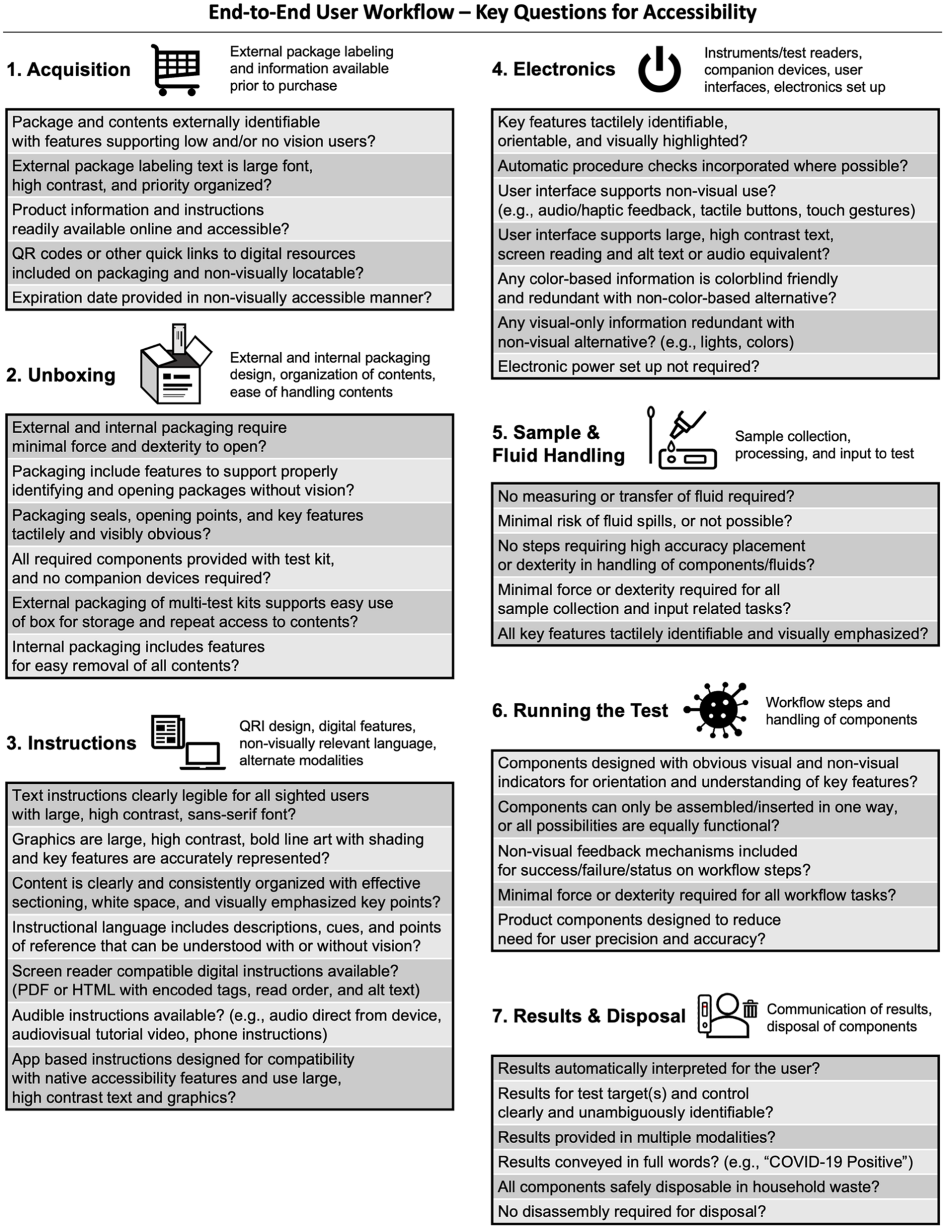
Key questions from the NIBIB RADx Tech III Program end-to-end accessibility assessment.

Funded projects underwent an initial end-to-end accessibility assessment between May 2023 and February 2024. Key assessment questions (Figure 3), derived from the *Best Practices for the Design of Accessible COVID-19 Home Tests* Technical Assistance Document (6), covered: (1) acquisition; (2) unboxing; (3) instructions; (4) electronics (where applicable); (5) sample and fluid handling; (6) running the test; and (7) results and disposal. Assessments were administered by program personnel, with ongoing support from accessibility consultants.

**Figure 4:**
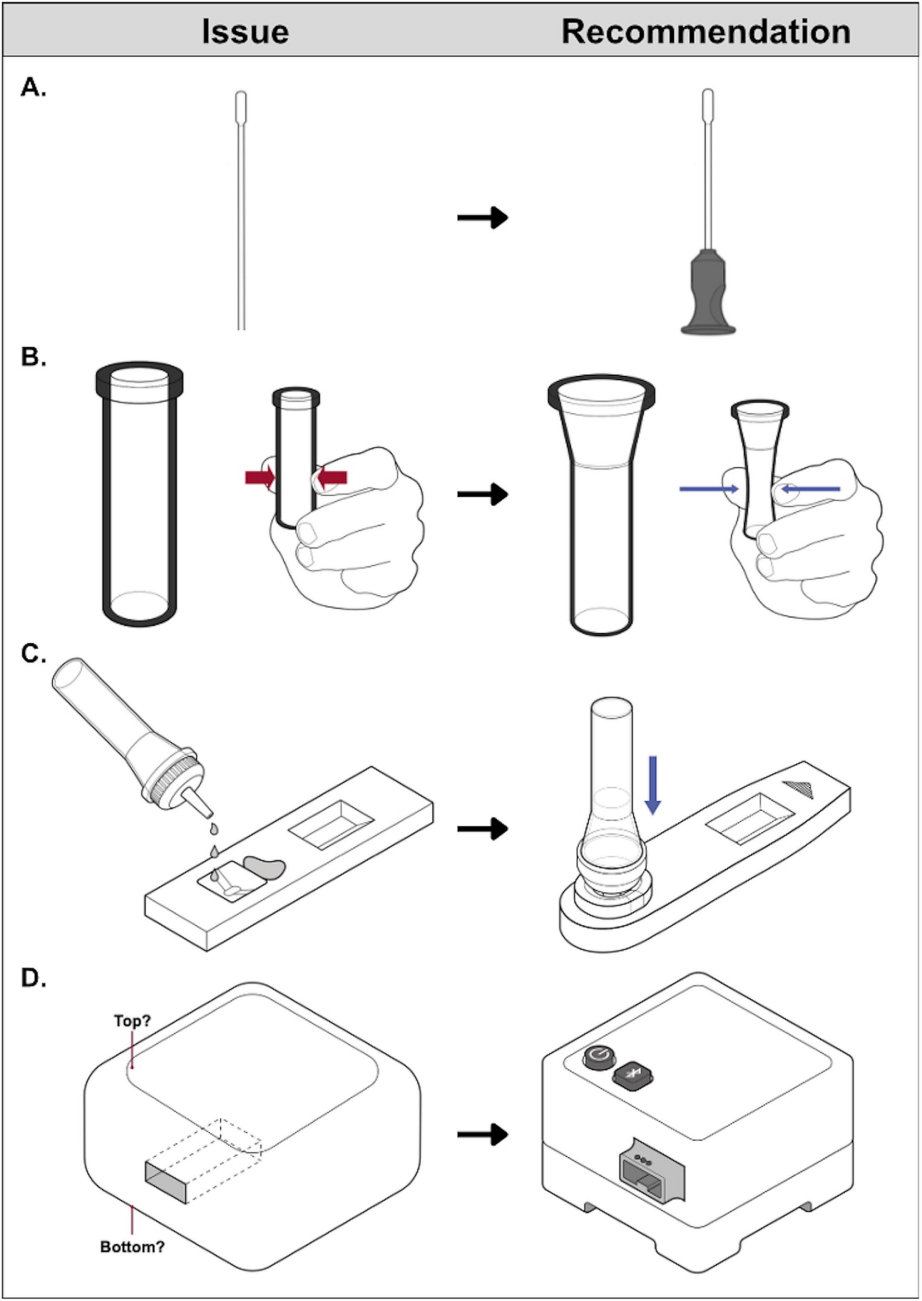
Examples of COVID-19 home test technological advances resulting from NIBIB’s RADx Tech III Program. Product features represent improvements to the accessibility of sample collection. **(A)** Adapted with permission from “Swab without handling feature” by U.S Access Board, sample processing. **(B)** Adapted with permission from “Thick-walled vial” and “Thin-walled vial” by U.S Access Board, fluid handling. **(C)** Adapted with permission from “Cassette without distinguishable well location” and “Cassette with docking feature” by U.S Access Board, and electronic test readers. **(D)** Adapted with permission from “Test reader without distinguishable features” and “Test reader with distinguishable features” by U.S Access Board.

Products redesigned as part of the RADx Tech III Program are not yet on the market but are expected to set a new standard for COVID-19 home test accessibility. Some swab shafts now incorporate a projecting feature to improve handling and tactile recognition of the swab’s orientation, reducing the likelihood that the swab packaging is opened at the wrong end, exposing the swab tip to touch contamination (Figure 4A). Manufacturers have widened fluid vial openings to ease swab insertion and made vial walls more pliable, so less force is required to squeeze vial walls inward to express sample from the swab tip or dispense fluid (Figure 4B). There has been a shift away from a rectilinear test cassette toward an asymmetrical cassette with tactilely distinct elements for ease of orientation. Several manufacturers have incorporated raised edges around the sample well to facilitate tactile recognition and fluid vial alignment with the well (Figure 4C). These modifications were found to help users differentiate the sample well from the results window and avoid spills during fluid transfer. Others have eliminated fluid transfer from the workflow entirely, typically by integrating fluids/vials and cassette components. Test readers–electronic devices that analyze test cassettes or samples and communicate test results (21)–now include: (1) distinguishing features for ease of orientation; (2) large, distinctly shaped, and sufficiently spaced buttons (Figure 4D); (3) large, high-contrast display screens and/or smartphone app connectivity for a secondary user interface; and (4) audio output in addition to visual output to convey instructions and/or status (e.g., cassette inserted properly, analysis underway, results available).

## 5 Discussion

### 5.1 Limitations and lessons learned

The NIBIB RADx Tech Accessibility Initiative’s focus on speed to market prevented resourcing through protracted competitive bidding processes. Staff succeeded in assembling a diverse pool of accessibility consultants and design firm talent through referrals and professional network/web queries, but this limitation is noteworthy. A competitive bidding process may have supported the recruitment of accessibility consultants with more balanced subpopulation expertise, reducing any design recommendation biases. Competitive bidding may also have enabled identification of design firms with experience designing devices incorporating accessible features fungible or adaptable to COVID-19 home tests. Professional network/web queries did not yield solid leads in this regard.

Additional takeaways related to resourcing behoove reflection by administrators of future accessibility initiatives. For instance, some SMEs living with disability or aging-related impairments were challenged and impassioned by the degree of inaccessibility encountered during test kit evaluations. SMEs who were able to maintain objectivity despite significant accessibility gaps contributed to the most productive dialog with manufacturers. Furthermore, each target user group–no vision, low vision, limited dexterity, and aging–conveyed distinct needs, and individuals had unique adaptive behaviors and compensatory strategies for bridging accessibility gaps. This makes achieving universal product accessibility complex and underscores the importance of reconciling multiple perspectives. For some product categories, a product portfolio, rather than a single product embodiment, may be necessary to meet heterogeneous needs.

Vetting of design firms that claimed a deep understanding of target user group needs revealed misconceptions. Many design firms considered disability simulation devices, like visual distortion goggles and arthritis simulation gloves, to be a sufficient substitute for involving individuals who live with vision, dexterity, and aging-related challenges. The stakeholder community repeatedly emphasized–and Initiative personnel observed firsthand–the importance of direct and continuous engagement with target users throughout the design process. SMEs representing target user groups have developed insight through lived experience that academics, even those with specialized training, are unable to develop on their own. Accessibility SMEs agreed that their central role in the RADx Tech Accessibility Initiative and Tech III program was a key differentiator. For many, this was the first time their needs, expectations, and desires had been deeply investigated as part of a product design/development process. One applauded the “user-centered approach, which ensured experts representing the disability groups served are a key component.” Another SME stated, “Building partnerships and expertise with companies and people with disabilities led to practical guidelines and impactful accessibility improvements.” A third remarked, “This team has shown a commitment to inclusivity far greater than I had expected.” A final SME provided an important reminder, “We have accomplished a lot. Many tests are much more accessible than they used to be, but there is still a long way to go. Our work will not be done until all at-home diagnostics are accessible to everyone.”

In contrast to the RADx Tech Accessibility Initiative, which focused on enhancements to mature technologies, RADx Tech III targeted early-stage technologies and was thus able to capitalize on front-end design flexibility to incorporate more impactful accessible product features (17). This highlights the importance of incorporating accessibility into the initial product design, especially for medical devices subject to regulatory control. Significant redesign post-regulatory approval is improbable because changes could impact product approval status and would likely require cost-prohibitive retooling and revalidation of manufacturing line equipment (22).

**Figure 5:**
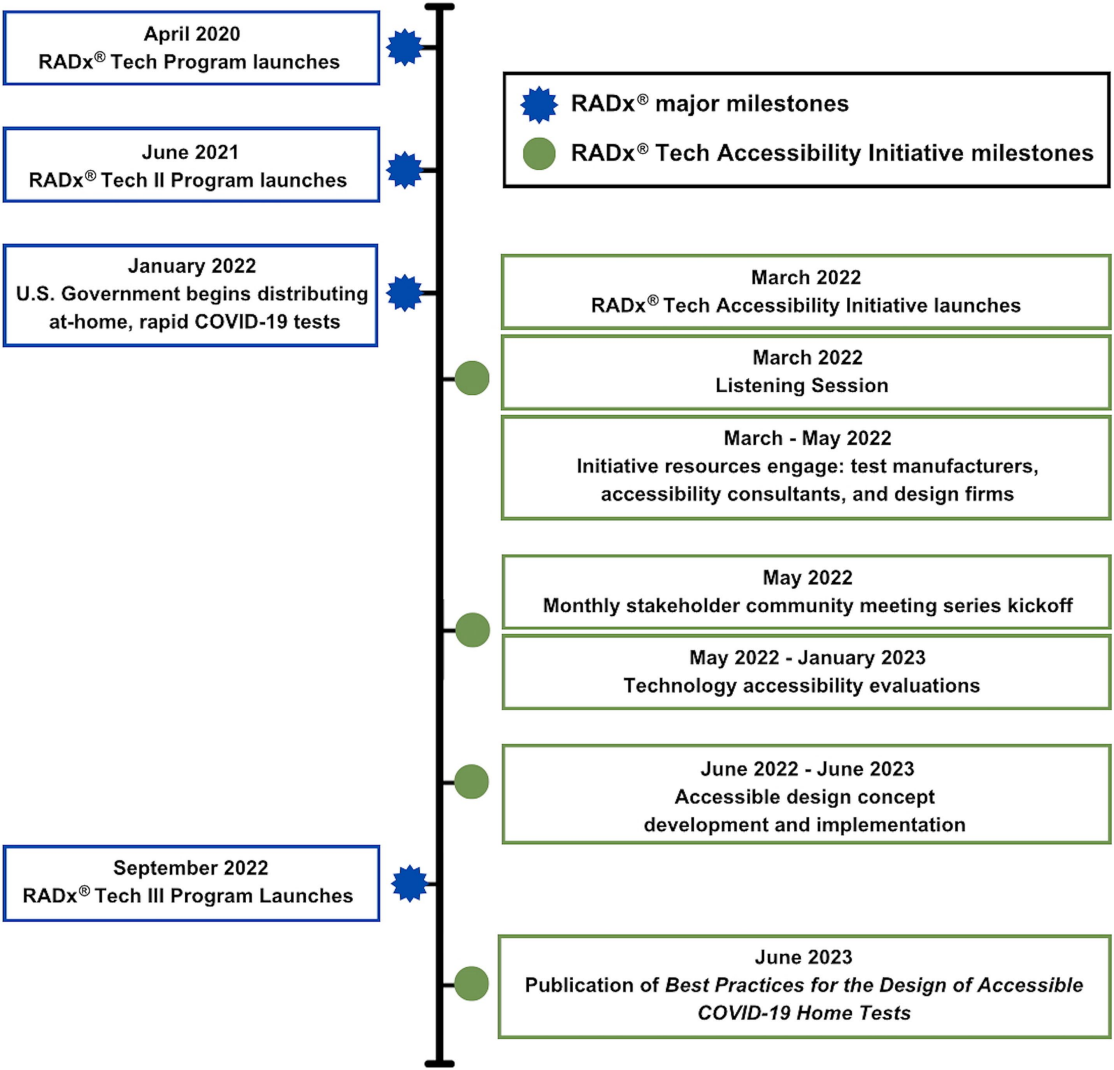
Timeline of NIBIB RADx Tech Accessibility Initiative milestones in context of umbrella RADx milestones.

The RADx Tech Accessibility Initiative had to assemble resources to construct a framework for accessible product development because standards and guidelines were nonexistent. The resulting framework, distilled in *Best Practices for the Design of Accessible COVID-19 Home Tests* (6), was well-received by most manufacturers. This suggests a receptiveness, at least in the IVD industry, to learning about how to improve product accessibility. If regulatory acceptance criteria for OTC products are updated to include accessibility (23), sweeping reforms will follow (Figure 5).

### 5.2 Cascading impacts

Design firms that participated in the Initiative have carried forward learnings to their current client engagements. A representative of one participating design firm explained, “Engagement with the RADx Tech Accessibility Initiative is helping us better advocate and create solutions for our clients that are usable by all populations. Through close collaboration with SMEs, we gained a wider lens and sharpened our human-centered design strategies, enabling more user independence and better use experiences. We are excited to challenge long-held assumptions, innovate to increase accessibility, and create real value for our clients and ultimately all users.” A representative of a second design firm added, “The RADx *Best Practices for the Design of Accessible COVID-19 Home Tests* document provides the design community with the first scientifically based repository of ergonomic design guidelines, strategies, and tactics applicable to all forms of in-home test kits.”

Some manufacturers of products that underwent accessibility evaluations and design sprints but whose implementation proposals did not receive NIBIB funding nonetheless proceeded with implementation. Also, due to the success of the RADx Tech Accessibility Initiative and follow-on RADx Tech III Program, accessibility considerations have permeated other programs under the RADx Tech umbrella. RADx Tech includes innovation funnels for diagnostic and monitoring technologies addressing health challenges beyond COVID-19. Focus areas include Influenza, Mpox, HIV, Hepatitis B and C, maternal health, fetal monitoring, and endometriosis (24). These other innovation funnels have leveraged HomeLab for accessibility evaluations like those conducted as part of the RADx Tech Accessibility Initiative, deployed accessibility SMEs for project-specific consults, and applied RADx Tech III’s end-to-end accessibility assessment (Figure 3) to funded technologies to identify and remedy accessibility gaps. As more RADx Tech products with accessible design features become commercially available, we expect competition to emulate the features consumers value most. The RADx Tech Accessibility Initiative and follow-on RADx Tech III Program accomplished the immediate aim of improving accessibility of COVID-19 home tests; but the bigger achievement was establishing a knowledge base and precedent for inclusive product design in the broader consumer medical device industry.

Mid-2024, when the U.S. Department of Health and Human Services (HHS) Administration for Strategic Preparedness and Response (ASPR) was preparing to distribute another round of COVID-19 home tests to the American public, the agency requested that the RADx Tech accessibility team members assess relative accessibility of shortlisted options. ASPR had already procured a large volume of the test RADx identified as the most accessible and worked with RADx to remediate digital instructions for accessibility, thus expanding COVID-19 test options for Americans living with disabilities (25). ASPR’s decision to seek advice from the RADx Tech accessibility team is a shining example of expanded reach.

## 6 Conclusion

Post pandemic, medical devices designated for OTC purchase and home use are becoming more prevalent (26). As the landscape evolves, commercialization of products accessible to all users–regardless of age, ability, or disability–becomes even more essential.

When the U.S. Government first began distributing rapid COVID-19 home tests in January 2022, these tests were inaccessible to many Americans with disabilities and aging-related impairments. The NIBIB’s RADx Tech Accessibility Initiative (March 2022–June 2023), and follow-on RADx Tech III Program (September 2022–present) have helped address the public health challenge of inaccessible COVID-19 home tests. Achievements include: (1) improvements to COVID-19 home tests accessibility with more advances in the commercial pipeline; (2) documentation of best practices for the design of accessible COVID-19 home tests; (3) a well-developed pool of experts in home test accessibility; and (4) a model for future national or international accessibility initiatives, especially those focused on consumer medical device innovation. The RADx Tech Accessibility Initiative and RADx Tech III Program detailed in this case study report represent novel public health interventions and encapsulate rich learnings for how to assemble and activate resources to advance product accessibility.

## Data Availability

The original contributions presented in the study are included in the article/supplementary material, further inquiries can be directed to the corresponding author.

## Footnotes

^1^This work was funded by the National Institutes of Health. Specific products are not mentioned to avoid the perception of product endorsement by the Government.
